# Learning as the unifying mechanism of psychedelic action

**DOI:** 10.1177/02698811251405683

**Published:** 2025-12-26

**Authors:** Alice Caulfield, Allan H. Young, Mitul Mehta

**Affiliations:** 1 Institute of Psychiatry, Psychology and Neuroscience, King’s College London, UK; 2 Division of Psychiatry, Department of Brain Sciences, Imperial College London, UK

**Keywords:** psychedelics, learning, critical period, sensitive period

## Abstract

Psychedelics are gaining attention as putative treatments for a range of psychiatric conditions, and evidence suggests that they produce sustained behavioural and clinical change. Enhanced learning is an emerging candidate mechanism mediating these sustained effects. This narrative review synthesises evidence across behavioural, neural and computational levels to examine the current evidence base for how psychedelics may alter learning mechanisms. We propose that viewing psychedelic mechanisms through the lens of learning unifies context-dependent outcomes, increased environmental sensitivity and neuroplastic change. We discuss how persistent changes in top-down and bottom-up information processing at a systems-level may account for both the therapeutic and adverse effects of psychedelics, and highlight a mechanistic convergence between such systems-level changes and the recently identified psychedelic-mediated reopening of critical learning periods. This systems-level framework may explain why psychedelic outcomes vary widely and hinge critically on the context: it is the learning environment, including psychological support and therapeutic insights, which shapes the lasting effect. If the post-psychedelic period is characterised by a vulnerable neuroplastic state in combination with increased environmental sensitivity, usually observed uniquely during childhood, this would offer a window of opportunity for revision of entrenched beliefs. Understanding these mechanisms has important translational relevance for the design and implementation of psychedelic-assisted psychotherapy.

## Introduction

The field of psychedelic drug research is deeply rooted in a rich cultural and political history. Psychedelics have been used throughout history by indigenous communities (George et al., 2020; Rätsch, 2005; Schultes, 1969). Despite this, they were later criminalised (Misuse of Drugs Act, 1971) and categorised as Schedule 1 substances in the UK (Howard et al., 2021), with similar regulations globally (Behera et al., 2024), under the assumption that they confer no medical benefits. Despite their criminalisation, there is a body of historical and more recent research suggesting they may have potential as treatments for a number of mental health conditions (Andersen et al., 2021). In addition, there is research suggesting they may produce sustained improvements in well-being (Aday et al., 2020).

Anecdotally, psychedelic experiences have been described as feeling ‘reborn’ (Grof, 2006), or experiencing a psychological ‘reset’ (Neitzke-Spruill et al., 2024). Suggestions that psychedelics may induce regressive behavioural states were noted as early as 1971 (Castellano, 1971); however, this idea received little empirical attention until recently. Recent research in experimental animals shows that psychedelics reopen critical periods (CPs) for social reward learning, underwritten by oxytocin-dependent meta-plasticity and structural remodelling of the extracellular matrix (ECM; Nardou et al., 2019, 2023). CPs are windows of heightened neuroplasticity observed during development, characterised by enhanced neural sensitivity to environmental input (Hensch, 2004). These findings not only add to the growing body of evidence showing that psychedelics induce neuroplasticity cascades (Weiss et al., 2025) but also suggest that this may be accompanied by a temporary change in behaviour, which, fascinatingly, shares qualitative similarities with a regression to earlier developmental stages (Grof, 2006; Neitzke-Spruill et al., 2024).

The neuroplasticity hypothesis has received significant attention in psychedelic research (for reviews, see Calder and Hasler, 2023; De Vos et al., 2021; Olson, 2022), and while evident in animals (Ly et al., 2018), a concrete demonstration of this in humans remains challenging. Despite accumulating neuroimaging evidence showing sustained neural changes following psychedelics in humans (Barrett et al., 2020; Doss et al., 2021; Madsen et al., 2021; Sampedro et al., 2017), the precise mechanisms underlying these changes remain poorly understood. Current studies often rely on proxies, such as changes in functional connectivity or behavioural flexibility, to infer neuroplastic changes; however, ‘neuroplasticity’ is not a singular process – arising from diverse mechanisms across different levels, from molecular signalling cascades, dendritic spine formation, synaptogenesis, to systems-level network reorganisations (Pearson-Fuhrhop et al., 2009). There is a critical knowledge gap in the existing literature: how may these (presumed) changes in human neuroplasticity translate into the observed lasting psychological and behavioural outcomes in humans?

Neuroplasticity alone does not guarantee adaptive outcomes; its functional relevance depends on the nature of the experiences and environmental inputs (Voss et al., 2017). We propose that learning acts as the mechanistic interface between cellular-level plasticity and higher-order psychological or behavioural outcomes. The link between these changes and neuroplastic changes remains unexplored in humans. Psychedelics are a particularly intriguing way to study this interaction, as they induce both intense experiences (Gashi et al., 2021) and neuroplasticity (Ly et al., 2018). We propose that a combination of psychedelic-induced neural malleability (see Ruffini et al. (2024) for support with this term) and enhanced environmental sensitivity (Fisher et al., 2024; Martin et al., 2024; Nardou et al., 2019; Šabanović et al., 2024) may catalyse learning. This framework could explain why outcomes vary so widely: it is the learning environment, including psychological support and therapeutic insights, which shapes the lasting effect.

The literature pertaining to serotonin-mediated learning enhancements has been reviewed previously (for review, see Carhart-Harris and Nutt, 2017), but otherwise, the body of evidence supporting psychedelic-induced enhancements of learning has attracted limited attention. More recently, the ‘pivotal mental state’ hypothesis (Brouwer and Carhart-Harris, 2021) has suggested that experiences can prime or trigger a mental state of rapid and deep learning, and that psychedelics hijack this inherent property of the human brain via 5-HT_2A_R agonism. In the present review, rather than focussing on conditions which enable learning, we discuss the influence of psychedelics on the learning process itself. A proposed (and measurable) mechanism for these learning enhancements is a change in top-down and bottom-up information flow (Makino, 2019). Indeed, this argument is made by one of the prevailing models of psychedelic action, the relaxed beliefs under psychedelics (REBUS) model, which proposes that a relaxation of priors (top-down processes) may enable revision of entrenched beliefs (Carhart-Harris and Friston, 2019). It is also supported by the thalamo-cortical gating model of psychedelic action, which liberates bottom-up sensory information flow (Vollenweider and Geyer, 2001). In light of the recent preclinical finding that psychedelics reopen CPs (Nardou et al., 2023), we propose that this hierarchical disruption may permit access to a juvenile-like enhanced learning capacity, where entrenched models are made permeable, and new associations can form more easily.

In this review, we will examine prevailing theories of psychedelic action and argue that a change in learning may be a unifying mechanism. We will briefly discuss the literature pertaining to changes in learning. We will propose an experimental human model to understand the interplay between learning and changes in hierarchical disruption, integrating computational and behavioural approaches. Such a perspective could deepen our understanding of how psychedelics catalyse sustained cognitive changes, with implications for our design of therapeutic interventions with psychedelics.

## Psychedelics and learning

The first neuroscientific suggestion that psychedelics alter learning mechanisms arose in the 1970s when lysergic acid diethylamide (LSD) was found to enhance classical conditioning of the nictitating membrane response in rabbits (Gimpl et al., 1979; Harvey et al., 1988, 2004; Romano et al., 2006, 2010; Welsh et al., 1998). These classical conditioning experiments were replicated, and the field expanded to test the effect of psychedelics on fear extinction, broadly showing enhanced fear extinction when psychedelics were administered before the extinction phase of the paradigm (Catlow et al., 2013; de la Fuente Revenga et al., 2021; Kelly et al., 2024; Werle et al., 2024; Woodburn et al., 2024). Enhancements in operant learning have also been observed, with enhanced acquisition of conditioned avoidance responses observed in shuttle box experiments (Bignami, 1972; Buchborn et al., 2014; Castellano, 1979; Izquierdo, 1975; Stoff et al., 1974). Evidence suggests that reversal learning may also be enhanced after psychedelics (Conn et al., 2024; King et al., 1974; Šabanović et al., 2024). While there is considerable variability in timings between task paradigms and drug administration, there is a growing body of evidence suggesting that psychedelics enhance learning across a range of paradigms.

Various mechanisms may underlie such enhancements in learning. At a receptor level, knockout and serotonin receptor antagonist studies show that psychedelic-mediated enhancements in fear extinction require 5-HT_2A/1A_ receptors (Kelly et al., 2024; Pędzich et al., 2022; Werle et al., 2024; Woodburn et al., 2024), and classical conditioning is dependent on 5-HT_2A_ receptors (Welsh et al., 1998). At a structural level, volume increases in sensory and associative cortical areas have been observed 1 day post 2,5-dimethoxy-4-iodoamphetamine (DOI) administration, with cognitive effects only emerging a week later, suggesting that these neuroplastic changes may provide a neural foundation to support behavioural change (Šabanović et al., 2024). Early studies suggest perceptual-level enhanced environmental sensitivity, with increased sensitivity to both conditioned and unconditioned stimuli observed in classical conditioning studies (Harvey et al., 1988; Gormezano and Harvey, 1980). An increase in environmental sensitivity is also suggested by several newer studies (Fisher et al., 2024; Kanen et al., 2023; Martin et al., 2024). Therefore, the recent finding that psychedelics reopen CPs (Nardou et al., 2023) presents a fascinating convergence of learning and neuroplasticity hypotheses, as CPs are characterised by increased environmental sensitivity, enhanced learning and enhanced neuroplasticity, all of which are inherently intertwined and context-dependent. The directionality of the relationship between these factors, which lie at the context, cognitive and neural level, respectively, remains to be established, and additionally, whether these factors may be different expressions of a common underlying neural substrate.

## Critical periods

CPs in the juvenile brain are discrete developmental phases of enhanced plasticity, where environmental input leads to irreversible changes to brain structure and function. Examples are imprinting and birdsong in animals (Hensch, 2004). More relevant to humans, sensitive periods (SPs) are similar windows where neural pathways become finely tuned by specific environmental inputs, and there is a shift in the biases or priorities to promote learning. Examples are language and pitch learning.

The neuroplastic mechanisms underwriting CPs are distinct from the adult brain, where plasticity is more constrained, characterised by limited structural reorganisation and primarily subthreshold synaptic changes (Morishita and Hensch, 2008). There are specific processes that regulate CP opening, maintenance and closure (Morishita and Hensch, 2008). During CPs, plasticity is marked by structural and functional changes, including neurogenesis, synaptic pruning, dendritic spine remodelling, and axonal reorganisation and network specialisation, leading to experience-dependent learning of new behaviours. This time-dependent dynamic plasticity arises from a precise excitatory-inhibitory (E-I) balance and permissive molecular conditions. Towards the end of the CP, fast-spiking parvalbumin-positive (PV+) inhibitory interneurons mature, and the E-I balance stabilises at a circuit level, with increased inhibitory tone. Molecular ‘brakes’ stabilise these established circuits at the end of the CP and constrain large-scale plasticity. A key example is the reorganisation of the ECM, including an increase in chondroitin sulphate proteoglycans, which promotes the formation of perineuronal nets (PNNs). These PNNs enwrap the PV+ GABAergic interneurons, which halt neuroplastic processes (Hübener and Bonhoeffer, 2014). Epigenetic modifications, such as increased histone deacetylase (HDAC) activity, suppress transcription of plasticity-related genes, such as *BDNF*, which is crucial in regulating experience-dependent plasticity during CPs (Anomal et al., 2013; Huang et al., 1999; Mui et al., 2018). Finally, the closure of CPs is marked by structural and functional stability (Knudsen, 2004; Marler et al., 1972). While this does not preclude future learning, it does make it more difficult, for example, as observed with language learning in humans.

Psychopharmacology studies have shown that many of these mechanisms, which regulate CPs are modulated by antidepressants or psychedelics, with corresponding behavioural-level changes suggestive of CP reopening (Gervain et al., 2013; Kobayashi et al., 2010; Nardou et al., 2019, 2023; Vetencourt et al., 2008). For example, chronic fluoxetine administration restores visual CP plasticity by reducing GABAergic inhibition in the visual cortex and increasing *BDNF* protein expression (Vetencourt et al., 2008). Subsequent research showed that this mechanism is dependent on reduced HDAC activity, which promotes histone acetylation at the BDNF promoter and facilitates the epigenetic remodelling required to restore CP-like plasticity (Vetencourt et al., 2011). Interestingly, sodium valproate (an *HDAC* inhibitor) was found to enhance absolute pitch learning in humans compared with placebo, suggesting that this molecular reopening of CPs may have meaningful behavioural effects in humans (Gervain et al., 2013). Ketamine also restores visual ocular dominance plasticity by targeting the molecular ‘brakes’ that close CPs. It disassembles the PNN covering of cells via microglial activity (Venturino et al., 2021) and downregulates Neuregulin 1 expression in PV+ interneurons (Grieco et al., 2020), both of which are known to constrain juvenile plasticity in the visual cortex. Recently, a range of psychedelics (ibogaine, LSD, psilocybin, 3,4-methylenedioxymethamphetamine and ketamine) were shown to reopen CPs through altered transcriptional regulation of the ECM in mice (Nardou et al., 2023). A shared downstream molecular mechanism among these drugs is implied as these transcriptional changes occur downstream of the drugs’ primary receptor targets (e.g. 5-HT_2A_, NMDA). While there is ample evidence of CP reopening in animals, measuring this in humans, at a level other than the behavioural level, is challenging. This is where a systems-level approach can be particularly useful.

For this review, we will use ‘SP’ to cover the critical – SP spectrum, as they both rely on the same neuronal learning mechanisms, and SPs are more clearly observed in humans. During SPs, learning is primarily driven by exposure to the environment, observed in rapid, unconstrained juvenile learning, driven by environmental statistics (Maye et al., 2002). Across development, the capacity for bottom-up input to induce neuroplasticity reduces, and learning moves away from being driven by passive exposure to becoming increasingly supervised by top-down processes, such as attention (Polley et al., 2006), prior knowledge and expectations (for review, see Kral and Eggermont (2007), White et al. (2013)). In other words, the systems-level context differs, and these systems-level changes may be measured in humans. As well as the above cellular-level changes supporting sensitive-period reopening as a mechanism of psychedelic action, emerging evidence suggests that similar systems-level changes may be observed in the psychedelic state (Alonso et al., 2015; Alamia et al., 2020; Rajpal et al., 2022). However, whether they specifically relate to enhanced learning capacity remains unknown.

## Top-down bottom-up disruption in psychedelics

Just as learning during SPs is dominated by bottom-up input with relatively weaker mature top-down control (White et al., 2013), increasing evidence suggests that psychedelics also disrupt the balance between top-down and bottom-up processes. Despite the accumulating evidence, whether such dynamics support enhanced learning remains unknown. Furthermore, it remains to be shown whether these shifts resemble the juvenile, bottom-up dominated learning observed during SPs. For ease, the top-down bottom-up shift will be referred to as TD-BU.

Changes in TD-BU dynamics have been described by the REBUS model, which proposes that psychedelics reduce the precision (inverse variance, or ‘certainty’) of high-level priors in the brain’s hierarchical generative model, through 5-HT_2A_R-mediated excitation of pyramidal neurons (Carhart-Harris and Friston, 2019). The REBUS model is rooted in the predictive coding framework, which conceptualises the brain as a hierarchical generative model that predicts the most likely cause of sensory input and refines its prior expectations through recurrent message passing across hierarchical levels, which suppresses precision-weighted prediction errors (PE; Friston, 2005). Prior distributions are carried by top-down processes, or backward connections and PEs are bottom-up signals passed up the hierarchy by forward connections, and refine top-down predictions according to their precision weighting (Friston, 2002, 2003, 2005; Friston et al., 2003). This gradual adjustment of our internal model over time corresponds to learning (Friston, 2003). The REBUS model posits that it is this reduction in prior precision weighting that permits revision of entrenched beliefs.

Empirical evidence supporting this model in humans comes from a variety of methodologies. Functional magnetic resonance imaging (MRI) has shown reduced activity under psilocybin in high-level association regions, such as the default mode network (Carhart-Harris et al., 2012), with a significantly decreased positive coupling between the posterior cingulate and medial prefrontal cortex observed. This is suggestive of a reduction of top-down connectivity from prefrontal to parietal regions, or an increase in bottom-up connectivity from parietal to prefrontal regions, or both. Electrophysiological studies also support this shift. Frequency-band analysis of resting state electroencephalograhy (EEG) data has shown diminished alpha-band travelling waves, which are considered to represent top-down control (Alamia and VanRullen, 2019), and increased bottom-up travelling waves, under the acute effects of dimethyltryptamine (DMT) (Alamia et al., 2020).

Transfer entropy (TE), which quantifies directed information transfer between neural regions (Schreiber, 2000), has been applied to EEG data obtained under the acute effects of ayahuasca. A decrease in anterior to posterior information transfer and an increase in posterior to anterior information transfer were observed, which is interpretable as an increase in bottom-up information flow, and a reduction in top-down control (Alonso et al., 2015). Interestingly, these changes correlated with the acute psychedelic effects and this pattern persisted, albeit non-significantly, up to the final measurement of 4 hours post-dose. While TE describes the directionality of information flow, it does not characterise the computational or functional significance of this, which is of critical relevance if we hypothesise that this may underwrite enhanced learning. Bayesian computational models offer a mechanistically plausible framework for assessing whether observed changes in information flow may arise from alterations in precision parameters. A recent study used Bayesian predictive processing modelling and magnetoencephalography to compare resting-state neural activity in healthy participants under LSD and ketamine, with neural activity from individuals with schizophrenia. The drug states were associated with a widespread overall reduction in TE across the brain (Rajpal et al., 2022), whereas individuals with schizophrenia exhibited increased TE, particularly from frontal to occipital regions. Modelling revealed that these apparently opposing changes could be unified by an increased weighting of sensory information over prior beliefs, by distinct mechanisms: a reduction in prior precision in the drug states and an increase in sensory precision in schizophrenia both of which correspond to a relative overweighting of bottom-up relative to top-down processing. These findings highlight the utility of Bayesian inference in further characterising electrophysiological measurements in a biologically plausible manner.

In addition to resting state data, task-based paradigms such as mismatch negativity (MMN) paradigms have been used to probe changes in PE processing. The MMN is a pre-attentive event-related potential elicited when a deviant stimulus is presented, which violates statistical regularities (Garrido et al., 2009). Within the predictive coding framework, the MMN is considered an index of sensory PE (Friston, 2005), and the process of resolving PEs through internal model updating corresponds to learning. Whereas MMN reductions are robustly observed in schizophrenia and ketamine studies (Rosburg and Kreitschmann-Andermahr, 2016; Umbricht and Krljes, 2005), the findings for psychedelics are inconsistent; with three studies reporting no change (Bravermanová et al., 2018; Schmidt et al., 2012; Umbricht et al., 2003), one reporting a reduction at sources S1 and S3 only (and may have been confounded by reductions in N1 amplitude, likely reflecting changes in early sensory processing; Heekeren et al., 2008), and two showing a diminished MMN (Duerler et al., 2022; Timmermann et al., 2018), one of which was a tactile (non-standard) mismatch paradigm (Duerler et al., 2022). When comparing DMT and ketamine, MMN attenuation was observed under ketamine but not DMT, while DMT uniquely reduced the N1 component (Heekeren et al., 2008). This suggests that despite converging downstream targets like BDNF and tropomyosin receptor kinase B (TrkB) (Casarotto et al., 2021; Moliner et al., 2023), there may be distinct changes when considering predictable and deviant sensory processing. Part of this discrepancy may relate to complex receptor binding profiles; for example, it is possible that the MMN reduction observed under LSD may be contributed to by D2 receptor partial agonism, as D2 antagonism (haloperidol) has been shown to increase the MMN (Kähkönen et al., 2001).

Beyond PEs, traditional MMN amplitude measures provide limited insight into the underlying computational mechanisms of predictive coding, such as precision weighting; however, dynamic causal modelling (DCM) may be used to characterise these mechanisms. DCM is a mechanistically plausible Bayesian framework that estimates the most likely neural architecture generating observed data, including EEG time series (Friston et al., 2003). For example, it may be applied to EEG data obtained from tasks including the MMN. It enables inference of effective connectivity (EC; extrinsic, between-region interactions), which estimates the influence one neural region has over another within a predefined network, and intrinsic connectivity (within-region), capturing processes like neural adaptation and PE formation (Garrido et al., 2009). DCM may also be used to estimate synaptic gain, a parameter linked to short-term plasticity and, in Bayesian terms, to precision at a neuronal level (Adams et al., 2016). LSD reduced the MMN to deviant stimuli and increased responses to standard stimuli, suggestive of reduced sensory adaptation (Timmermann et al., 2018). DCM estimated that these effects were linked to reduced intrinsic connectivity in the primary auditory cortex and attenuated top-down (backward) connectivity, suggesting a heightened sensitivity towards external stimuli and a reduction in top-down influence (Timmermann et al., 2018). In addition, DCM of functional MRI (fMRI) data showed increased EC from the thalamus to the posterior cingulate cortex under LSD, in a manner which depended on 5HT-_2A_R activation (Preller et al., 2019), supporting reduced thalamic gating and a liberation of bottom-up information flow.

In summary, neuroimaging studies suggest that there is a shift in TD-BU, but the functional relevance of this requires further exploration. Literature regarding the behavioural correlates of this is extremely limited. Behavioural studies have attempted to probe TD-BU shifts using alternative approaches, such as reinforcement learning (RL). For example, LSD increased (reinforcement) learning rates for both rewards and punishments, particularly for reward outcomes, in a probabilistic reversal learning task (Kanen et al., 2023). In the RL framework, learning rates weight the PE. It may therefore be considered analogous to Bayesian precision weighting of PEs (Mathys et al., 2011). Indeed, the authors suggest that the enhanced sensitivity to PEs observed from the increased learning rates is naturally consistent with a down-weighting of priors. Whether such changes in these learning parameters can be interpreted as being consistent with SP-like effects remains unclear. Furthermore, studies of learning rate changes across human development report mixed results, likely due to task-specific and cross-sectional designs (for review, see Nussenbaum and Hartley, 2019). An approach using a combination of RL and Bayesian approaches, with SP learning paradigms, may be a useful avenue for future research. For example, a study combining a behavioural measurement which has evidence of a SP (such as absolute pitch learning), with DCM of EEG time series under drug versus non-drug conditions is a protocol bringing together sensitive approaches to address the question of SP reopening and enhanced learning in humans.

## Persisting hierarchical disruption may support enhanced learning

Several lines of evidence suggest that psychedelics shift the balance from top-down to bottom-up processing acutely; however, a critical outstanding question is whether this is sustained. Recent data suggest that these hierarchical disruptions may indeed persist in the post-acute phase. A reanalysis of psilocybin fMRI data using a whole-brain EC model demonstrated sustained decreases in top-down information flow and increases in bottom-up, 1 month after administration (Pasquini et al., 2024). Interestingly, these EC changes were associated with the distribution of 5-HT_2A_ and D_2_ receptors, respectively, suggesting there may be distinct neuromodulatory contributions to hierarchical processing.

A critical question remains regarding the functional relevance of this shift. If these shifts in hierarchical processing are in fact akin to SPs, there may be a window of opportunity not typically accessible to adults, characterised by a unique sensitivity to the environment and supportive of environmental learning. This may explain why set and setting are so critical in the psychedelic experience. In Bayesian terms, a combination of sustained reduced prior precision, with an enhanced sensitivity to bottom-up PEs, would permit new learning which (if handled correctly) could be hypothesised to underwrite therapeutic change.

Through a computational lens, post-acute classical psychedelic-mediated environmental sensitivity remains unexplored in humans; however, this has been examined using ketamine. In treatment-resistant depression patients, an enhanced sensitivity to PEs (measured by a greater MMN) was observed in the post-acute phase (measured 4 hours after ketamine infusion), which is in contrast to the typically diminished MMN observed with acute ketamine (Rosburg and Kreitschmann-Andermahr, 2016). Interestingly, DCM revealed an increase in forward (bottom-up) connectivity, which correlated with antidepressant response (Sumner et al., 2020). This is suggestive of an increase in environmental sensitivity, with a presumed acceleration of generative model updating. However, whether these observations hold for non-depressed populations remains unknown. Nonetheless, these findings are particularly intriguing given that ketamine has also been shown to reopen CPs in preclinical research (Nardou et al., 2023). Whether classical psychedelics similarly induce a window of enhanced sensitivity to PEs, and the behavioural relevance of this, remains to be established.

Preclinical research offers several lines of evidence suggesting that psychedelics enhance environmental sensitivity. For example, enhanced sensitivity to conditioned and unconditioned cues has been observed in classical conditioning experiments in rabbits (Gormezano and Harvey, 1980; Gormezano et al., 1980; Harvey et al., 1988). Enhanced dopamine signalling was observed in rats administered the psychedelic DOI, in response to both rewards themselves and, unusually, fully predictable proximal reward cues (and importantly, this was independent of changes in reward value; Martin et al., 2024). This suggests that DOI may be amplifying PE signalling, or perhaps relatedly, may confer an enhanced perception of novelty or surprise. Another study found that DOI increased the rate of adaptation to novel task reversals in a two-step probabilistic reversal learning task in mice (Šabanović et al., 2024). This improvement was only observed when the task reversal was introduced 1 week post-treatment (and not 1 day post-treatment); in other words, the drug had no immediate effects on reversal learning, and the improvement was dependent on task experience during the week following drug administration. Without task experience, DOI conferred no measured cognitive benefits, implying that the context (experiencing the task) during the window after DOI was essential (Šabanović et al., 2024). A new (and unusual) learning strategy was observed, where mice began learning from reward omissions, suggestive of a heightened sensitivity to previously overlooked environmental stimuli.

These various findings point to a shared mechanism of increased environmental sensitivity and may be unified by a shift in TD-BU processing. Together, these results suggest that psychedelics transiently relax top-down constraints and amplify bottom-up PE signalling across neural, computational and behavioural levels.

## Proposing a model to measure SP reopening in humans

Given the challenges of accessing molecular and synaptic plasticity mechanisms in humans, systems-level and computational approaches are increasingly useful. DCM may be used to approximate neuroplasticity changes by measuring functional coupling changes within neural circuits as a function of environmental experience. EC permits modelling of causal interactions between brain regions (Friston, 1994), and if combined with behavioural models of learning, will help to understand how top-down and bottom-up information processing may change and interact with learning after psychedelic administration. A systems-level approach is of particular interest given the recent finding that psychedelics induce neuroplasticity and antidepressant-like behaviour, independently of the 5-HT_2A_R, by binding to the BDNF receptor TrkB in mice (Moliner et al., 2023). Ketamine and conventional antidepressants also bind to TrkB to potentiate BDNF signalling (Casarotto et al., 2021), implying a fascinating convergence suggestive of broader biological or systems-level mechanisms that transcend traditional receptor-centric models.

We propose that psychedelics lead to a shift in TD-BU, which is transiently sustained after the acute effects have subsided. This hierarchical disruption would support enhanced learning from the environment, and this period of heightened neural and behavioural malleability may provide an optimal timeframe for therapeutic interventions, where environmental enrichment and guided learning could maximise the therapeutic benefits of psychedelics. Alternatively, it may provide a window during which insights gained during psychedelic therapy may be integrated into daily life. It seems likely that this period is underwritten by neuroplastic changes in humans, although this remains speculative. While measuring neuroplastic changes is an actively evolving area of psychedelic research in humans, these neuroplastic changes must be accompanied by a measurable behavioural change for adequate mechanistic interpretation. While studies have measured changes in TD-BU in humans, as reviewed here, none have attempted to link this with changes in learning. This is a critical area for future research, and a study design that relates changes in TD-BU, for example, through DCM applied to neuroimaging data, with a learning paradigm which approximates sensitive-period learning, would achieve this. Such an approach would be critical to infer whether psychedelics truly reopen SPs in humans or simply enhance general plasticity mechanisms. Possible targets for sensitive learning paradigms in humans would include language learning or absolute pitch learning, as has been investigated using valproate (Gervain et al., 2013). Measuring TD-BU changes in a control group that receives the psychedelic but does not receive task training would help understand how TD-BU changes interact with learning, and whether this relationship is bidirectional.

In summary, through the use of a learning task (which has evidence of a SP), we can understand whether psychedelics reopen SP learning, and through DCM, we can understand whether there is a disruption to information flow on a systems level, which is akin to SP reopening. Importantly, this does not preclude other mechanisms, and future research should seek to clarify the associated behavioural correlates and changes in hierarchical processing.

## Conclusion

It is established that psychedelics acutely disrupt the balance between top-down and bottom-up processing, with supportive evidence coming from both resting and task-based neuroimaging studies, complemented by both Bayesian and RL models. This shift is central to REBUS, the prevailing model of psychedelic action. Recent evidence suggests that these changes may persist beyond the acute psychedelic state, and that psychedelics may reopen CPs of learning, which are themselves characterised by bottom-up, environmentally driven learning underwritten by comparatively unconstrained neuroplasticity.

This evidence is complemented by a history of psychedelic-mediated enhancements in learning, and we propose that viewing psychedelic mechanisms through the lens of learning unifies cognitive changes, increased environmental sensitivity and neuroplastic change. It is plausible that in the post-psychedelic period, a vulnerable neuroplastic state in combination with increased environmental sensitivity, usually observed uniquely during childhood, would offer a window of opportunity for revision of entrenched beliefs.

The current psychedelic-assisted psychotherapy model incorporates unstructured psychotherapy during the acute drug experience, with ensuing integration sessions. If there is indeed a window of greater environmental sensitivity after psychedelics, then firstly, this period should be factored into the model (in other words, going back to a stressful domestic or employment setting may be harmful), and secondly, therapeutic interventions during this window may be enhanced. It may even be possible to enhance the learning of skills which would typically be acquired during early developmental phases. While this remains to be clarified in humans, there is a clear need for improved mechanistic characterisation of the post-acute period.
